# New molecular pathways in angiogenesis

**DOI:** 10.1038/sj.bjc.6601107

**Published:** 2003-07-15

**Authors:** D C Sullivan, R Bicknell

**Affiliations:** 1 Molecular Angiogenesis Laboratory, Cancer Research UK, Institute of Molecular Medicine, University of Oxford, John Radcliffe Hospital, Oxford OX3 9DS, UK

## Abstract

Angiogenesis has developed into a major area of cancer research. Recently, several newly identified signalling pathways have been shown to play a role in both normal and pathological (including tumour) angiogenesis. Several of the molecules involved in these pathways have potential as novel anti-cancer therapeutic targets including members of the ephrin/Eph receptor, Notch/delta, sprouty, hedgehog and roundabout/slit families. These developments are reviewed.

Angiogenesis describes the formation of new blood vessels from the existing vasculature, a process that is relatively rare in the healthy human adult, occurring only during the female menstrual cycle and in wound healing. In contrast, angiogenesis occurs in many pathologies including diabetic retinopathy, arthritis, atherosclerosis, psoriasis and tumour growth. It has long been postulated that abrogation of angiogenesis could be an effective anticancer strategy; however, in order to be able to design effective antiangiogenic treatments, we must first understand how new blood vessels form. The inception of the vascular system occurs early in mammalian development with the differentiation and aggregation of angiogenic precursors in the embryo culminating in the blood islands of the visceral yolk sac. The early blood vessels of the embryo and yolk sac develop by aggregation of angioblasts that *de novo* create a primitive network of simple endothelial tubes – the process of vasculogenesis. Extensive research into the molecular mechanisms involved in vessel formation has identified proangiogenic factors such as vascular endothelial growth factor (VEGF) and the angiopoietins, together with antiangiogenic factors such as the thrombospondins and transcription factors leading to their expression like Id1 (reviewed in Bikfalvi and Bicknell, 2002). Two recent developments in the field have been the delineation of the mechanism by which hypoxia acts as a proangiogenic stimulus via the oxygen-sensing prolyl hydroxylase and hypoxia-inducible factor-*α* and the identification of several novel extracellular angiogenic signalling pathways. The latter include the ephrin/Eph receptor, notch/delta, hedgehog, sprouty and slit/roundabout families (Figure 1

**Figure 1:**
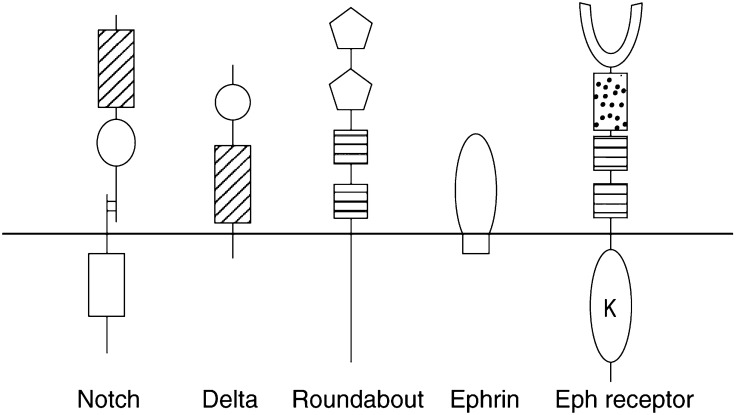
Novel signalling molecules on the endothelial surface involved in angiogenesis. IgG domain (pentagon), fibronectin-like domain (horizontal hatched box), EGF-like repeats (diagonal hatched box), cysteine-rich region (spotted box), DSL domain (circle) and tyrosine kinase domain (K). Examples known to be present on tumour endothelium are Notch4, Delta4, magic roundabout, ephrin B2 and EphB3 and -B4.

). Here, we review the role of these signalling pathways in angiogenesis and point out where appropriate as to how they could be utilised to develop new anticancer strategies.

## EPHRINS, THE EPH TYROSINE KINASE RECEPTORS AND ANGIOGENESIS: VALIDATED ANTITUMOUR TARGETS

The ephrin ligands and Eph receptors comprise an increasingly studied family of signalling molecules originally identified some 13 years ago. Like other families of signalling molecules, they are not restricted to the endothelium, but are found in numerous cell types. Also, like the notch/delta family described later, the ephrin ligands and their Eph receptors are both membrane-bound molecules. The ephrin ligands are divided into A and B type molecules that are distinguished by the way in which they are anchored in the plasma membrane. Thus, the ephrin A ligands are tethered to the outer plasma membrane via a glycosylphosphatidylinositol (GPI) anchor, whereas the ephrin B ligands are inserted into the plasma membrane via a transmembrane region followed by a conserved cytoplasmic domain. The ephrins bind to two families of transmembrane EphA and EphB tyrosine kinase receptors. While the A-type ephrins preferentially bind to the EphA receptors and the B-type ephrins to the EphB receptors, within each subclass, considerable promiscuity of receptor binding has been demonstrated.

Several ephrins and Eph receptors have been found in the vascular endothelium. These include ephrin A1 that plays a role in the inflammatory angiogenesis induced by tumour necrosis factor-*α* (Pandey *et al*, 1995) and ephrin B1 that promotes endothelial capillary-like assembly and attachment *in vivo* (Stein *et al*, 1998). Ephrin B2 and the eph receptors EphB3 and EphB4 are also present in vascular endothelium. The involvement of ephrin ligands and ephrin receptors in vascular development is already so extensive that it is not feasible to review all the studies here. Interested readers are referred to recent reviews (Adams, 2002; Cheng *et al*, 2002a). A clear role for ephrins in normal vascular development presaged a role in pathological tumour angiogenesis.

## EPHRIN LIGANDS AND EPH RECEPTORS IN TUMOUR ANGIOGENESIS

A detailed study by Ogawa *et al* (2000) showed that the ephrin A1 ligand and its EphA2 receptor are expressed in tumour angiogenesis. Thus, double immunostaining of endothelial cells for CD34 showed ephrin A1 and its EphA2 receptor to be expressed throughout the endothelium in mouse xenografts of human MDA435 and K1767 Kaposi sarcoma cells and in the vasculature of human cancers. A dominant-negative EphA2 receptor blocked the formation of capillary endothelial tubes *in vitro*. Further studies have shown that soluble EphA2-Fc and EphA3-Fc receptor constructs inhibit tumour angiogenesis and growth *in vivo* (Brantley *et al*, 2002), providing the first functional evidence for EphA receptor regulation of tumour angiogenesis. Recent mechanistic studies have shown that blockade of the EphA receptor specifically inhibits VEGF-induced angiogenesis (Cheng *et al*, 2002b). This activity is not restricted to members of the A subclass as Martiny-Baron *et al* (2001) have reported similar effects with the soluble extracellular domains of the ephrinB2 and EphB4 receptors. There is clearly much work to do, but it appears that abrogation of the function of both the A and B class ephrins may provide novel antiangiogenic and antitumour activites.

## NOTCH SIGNALLING IN ANGIOGENESIS

The construction of a multicellular organism with characteristic size and shape from a single cell requires complex coordinated gene action to direct the fate of each cell. An organism will often use the same signalling pathway within different cellular contexts to achieve unique developmental objectives. Originally characterised in *Drosophila* (Artavanis-Tsakonas *et al*, 1999), the Notch receptor and its ligands Delta/Serrate are one example of such a signalling system that plays many roles throughout development, as well as affecting cell cycle progression and apoptosis.

The complexity of the Notch system in vertebrates is illustrated by the existence of multiple Notch receptors and ligands, each having a distinct expression profile. Thus, there are four Notch receptors in man, Notch 1–4 and five ligands, Jagged1 and 2 and Delta (Dll)1, -3 and -4. The activation of Notch upon ligand binding is accompanied by proteolytic processing that releases an intracellular domain of Notch (NICD) from the membrane. The intracellular domain then translocates to the nucleus where it associates with the CSL family of DNA-binding proteins to form a transcription factor. This complex initiates the transcription of a set of target genes, including the Enhancer of Split group E(spl) and others; in this way Notch functions as a membrane-bound transcription factor. That Notch is active during human vascular development is illustrated by the cerebral disorder, cerebral autosomal-dominant arteriopathy with subcortical infarcts and leucoencephalopathy (CADASIL), which arises from a mutation in Notch3. CADASIL is characterised by stroke, vascular dementia and arteriopathy of cerebral arterioles.

Direct evidence from several other studies has indicated a role for Notch signalling during vascular development as well as during maintenance of vessel homeostasis in the adult. When an activated form of the Notch4 protein is under the regulation of the Flk1 (VEGFR) promoter in mice during embryonic development, the expression of this activated Notch results in growth and developmental delay and embryonic lethality at embryonic day 10 (Uyttendaele *et al*, 2001). These mice had fewer vascular networks and those that did form were disorganised with less small vessels present. A similar phenotype was observed when *Jagged1* was inactivated in the mouse embryo (Xue *et al*, 1999). Finally, haemorrhage was observed in *delta1* mutants (Hrabe de Angelis *et al*, 1997). In fact, both increases and decreases in Notch signalling in mice produce similar vascular phenotypes, suggesting that the level of signalling is critical for optimal blood vessel development. The presence of a misformed vasculature in such mice suggests that Notch signalling does not function early in vasculogenesis, but rather regulates subsequent remodelling events that pattern the vascular network.

The expression of all currently known Notch ligands and receptors in the developing vasculature has been examined (Villa *et al*, 2001). It was found that Notch1, -3 and -4, Delta4, Jagged1 and -2 are all expressed in arteries but not all are expressed by veins. This pattern of restricted expression to arterial blood vessels is similar to that of ephrin B2. It was also found that Notch4 and Delta4 are the only receptor and ligand in the family that are present in capillaries. These observations implicate the Notch pathway in the later stages of vascular development, but not the initial specification of the lineage. There is evidence that the Notch pathway is involved in responses to vascular injury since Jagged1, -2 and Notch1, -3 and –4 expression is increased in injured vascular cells (Lindner *et al*, 2001). This study also demonstrated that cell–cell contacts and adhesion plaques are potential targets of Jagged/Notch signalling because cadherin-mediated intercellular junctions as well as focal adhesions were modified in cells transfected with Jagged1. Thus, Jagged regulation of cell–cell and cell–matrix interactions may contribute to the control of cell migration in situations of tissue remodelling *in vitro*. Evidence of the angiogenic signalling that controls Notch and ligand gene expression have recently appeared (Liu *et al*, 2003). Thus, it was shown that VEGF, but not FGF, induces Notch1 and Delta4 expression in human arterial endothelial cells. Delta4 is of particular interest to those studying tumour angiogenesis as it is absent or poorly expressed in adult tissues but shows high expression in the vasculature of xenografted human tumours and in endogenous human tumours (Mailhos *et al*, 2001). Expression of Delta4 on the tumour vasculature is no doubt a result of it being one of the recently identified hypoxically induced endothelial specific genes (Mailhos *et al*, 2001). This expression pattern identifies Delta4 (along with magic roundabout, see later) as an ideal molecule with which to target the tumour vasculature. The potential of the Notch/Delta system for therapy is as yet largely unexplored territory.

## HEDGEHOG SIGNALLING IN ANGIOGENESIS

Hedgehog proteins have been shown to act as morphogens in numerous different tissues during embryonic development. They are 19 kDa proteins that interact with heparin on the cell surface through an N-terminal basic domain and are tethered to the surface through cholesterol and fatty acyl modification. Hedgehog signalling is crucial for the formation of limb, lung, gut, hair follicles and bone. There are three human homologues of the *Drosophila* hedgehog gene: sonic hedgehog (Shh), desert hedgehog (Dhh) and Indian hedgehog (Ihh). Of these, Shh is the most widely expressed during development and lack of Shh is embryonically lethal with multiple defects in early to mid-gestation. Ihh is less widely expressed and mice deficient in Ihh are able to survive until late gestation but die due to skeletal and gut defects. Dhh-deficient mice are viable but display peripheral nerve and male fertility defects.

Signalling by all three Hedgehog proteins occurs through interaction with the Patched1 receptor, which then activates the transcription factors Gli1, Gli2 and Gli3. The downstream targets of the Gli gene products include both patched and Gli themselves, thus patched and Gli are both components and targets of the Hh signalling pathway.

There is increasing evidence of a role for Hh signalling in angiogenesis. For example, hypervascularisation of the neuroectoderm is seen following transgenic overexpression of Shh in the dorsal neural tube of zebrafish. As with Notch signalling, it appears that both up- and downregulation of Hh proteins result in vascular defects. These observations clearly suggest a role for Hh signalling in angiogenesis but do not actually prove a direct role for the Hh protein. While Shh has been shown to have an indirect role in angiogenesis by acting upstream of angiogenic factors (Pola *et al*, 2001), it has also been shown to be a potent angiogenic agent *in vivo*. Thus, when Shh is administered to aged mice it induced new vessel growth in ischaemic hind limbs. The Shh-induced vessels showed a characteristically large diameter. Despite this, Shh had no effect *in vitro* on endothelial-cell migration or proliferation but did induce the expression of proangiogenic VEGF and angiopoietins-1 and -2 from interstitial mesenchymal cells. The indirect nature of hedgehog signalling has been confirmed in zebrafish (Lawson *et al*, 2002). Thus, it was shown that zebrafish embryos lacking Shh activity fail to undergo arterial differentiation, as defined by the expression of artery-specific markers such as *ephrin-B2a*. However, injection of mRNA encoding Shh into the zebrafish could induce ectopic vascular expression of *ephrin-B2a* as did the injection of *vegf* mRNA (Lawson *et al*, 2002). The loss of arterial marker gene expression and ectopic *flt4* transcripts in the dorsal aorta is similar to that of embryos with defective Notch signalling, suggesting that the *vegf* and the Notch pathway act in a common signalling system to induce arterial differentiation. Notch in the absence of *vegf* is able to rescue *ephrin-B2a* expression, providing evidence for such a common cascade. It seems that Shh might have a role in the spatial–temporal production of angiogenic growth factors during embryonic and postnatal angiogenesis, working upstream of *vegf*, which in turn operates upstream of Notch.

## SPROUTY AND ANGIOGENESIS

Sprouty (Spry) was first identified in *Drosophila* as an inhibitor of fibroblast growth factor (FGF) signalling during tracheal development. It is an intracellular protein, localised to the inner leaflet of the plasma membrane by a cysteine-rich domain. In *Drosophila*, Spry is expressed at the tips of growing primary branches of the tracheal system, in the eye imaginal disc, the embryonic chordotonal organ precursors and in the midline glia. There are four isoforms of Spry in mammals, each having a highly conserved C terminus but variable N terminus. They are all expressed in restricted patterns in the embryo in early development, showing dose correlation with sites of FGF signalling suggesting that Spry proteins may function as negative regulators of FGF signalling during vertebrate development as well as in *Drosophila*. In fact, a decrease in Spry2 expression in the mouse results in increased lung branching morphogenesis. As with tracheal development, the process of angiogenesis requires receptor tyrosine kinase signals. In addition, FGF, VEGF, platelet-derived growth factor, ephrin B and Tie-2 are all components of both tracheal and blood vessel development. In view of these many similarities between *Drosophila* trachea development and mammalian angiogenesis in terms of gene function, therefore, it was anticipated that Spry would also have a role in angiogenesis.

Direct evidence for a role for Spry in angiogenesis comes from a study in which the mouse Spry4 was overexpressed in the developing endothelium of a mouse embryo using an adenoviral vector (Lee *et al*, 2001). Microangiography was used to examine the effect of mSpry overexpression in the developing embryo, a procedure where a phenol red dye was injected into the vitelline artery of the embryo and the vessels in the embryo were then visualised by the circulating dye. It was found that embryos expressing mSpry4 had decreased sprouting of smaller vessels from the larger ones. By embryo whole mount staining with an anti-PECAM antibody, it was found that mSpry4 injection resulted in the development of a primitive vasculature with poor branching and minimal sprouting of vessels. Furthermore, 24 h after injection, the hearts of the mSpry4 expressing embryos were beating, but were incompletely developed. When HUVEC *in vitro* were transfected with mSpry4 there was a decrease in cell migration and cell cycle arrest at the G1/S phase with no apoptosis. The action of Spry4 appears to be via receptor tyrosine kinase pathways since there was a reduction in both basal and bFGF or VEGF-induced MAPK phosphorylation in Spry4-expressing cells. MAPK signalling is involved in the regulation of proliferation, migration and differentiation during angiogenesis and the inhibition of tyrosine kinase-stimulated MAP kinase activation probably accounts for the observed cell cycle arrest. To date it is not known whether the other sprouty proteins have similar antiangiogenic properties since the mode of action of sprouty in angiogenesis is very much in its infancy.

## ROUNDABOUTS AND SLITS IN ANGIOGENESIS

Roundabouts and slits comprise a family of signalling molecules thought to be restricted to cells of neuronal lineage. Roundabout was so named because of the neuronal phenotype arising from its deletion in *Drosophila*. The slits and roundabouts are involved in axon guidance where the three known human slits and two roundabout receptors mediate a repulsive signal. The recent identification of magic roundabout (ROBO4), a novel roundabout receptor restricted to endothelial cells was unexpected (Huminiecki and Bicknell, 2000). *In situ* analysis has shown magic roundabout to be absent from adult tissues, but strongly expressed on the vasculature of tumours including those of the brain, bladder and colon metastasised to the liver (Huminiecki *et al*, 2002). These observations identify magic roundabout as a promising therapeutic target.

## CONCLUSIONS

It is arguably true to say that few researchers in the field of angiogenesis expected the growth of new blood vessels to be a simple event, nevertheless the molecular complexity of the process and the number of pathways involved has been a surprise. A significant outcome of this complexity has been the pleasing number of potential new targets available for therapeutic intervention. Studies of antiangiogenesis and vascular targeting are clearly at an exciting stage.
